# Determination of Mechanical Properties of Sand-Coated Carbon Fiber Reinforced Polymer (CFRP) Rebar

**DOI:** 10.3390/polym15092186

**Published:** 2023-05-04

**Authors:** Hyun-Do Yun, Sun-Hee Kim, Wonchang Choi

**Affiliations:** 1 Department of Architectural Engineering, Chungnam National University, Daejeon 305-764, Republic of Korea; 2 Department of Architectural Engineering, Gachon University, Seongnam-si 13120, Republic of Korea

**Keywords:** tensile strength, compressive strength, elastic modulus, shear strength, sand-coated carbon fiber-reinforced polymer (CFRP)

## Abstract

This experimental study investigates the fundamental mechanical characteristics of the carbon fiber-reinforced polymer (CFRP) bars, including the tensile strength, compressive strength, shear strength, and modulus of elasticity of the CFRP bar. The properties need to be accurately determined to understand the behavior of the concrete structures reinforced with CFRP rebars. The CFRP rebar was coated with sand to enhance the adhesive strength of the concrete. Three diameters of CFRP rebar (D10, D12, and D16) were considered in accordance with ASTM provisions. A coefficient, i.e., the ratio of shear strength to tensile strength, was employed to predict the tensile strength of the CFRP rebar specimens. The test results confirm that the tensile strength of CFRP rebar is dependent on its diameter due to the shear lag effect. A coefficient in the range of 0.17 to 0.2 can be used to predict the tensile strength of CFRP rebar using shear strength.

## 1. Introduction

For decades, research into composite materials has explored the feasibility of a replacement material for conventional reinforcement in concrete structures. In Japan, Europe, Canada, and the United States, research for alternative materials for conventional steel rebar has been actively conducted, among them, fiber-reinforced polymer (FRP) composites are employed as an alternative. FRP materials are widely used in the construction industry due to their superior mechanical and physical advantages such as high chemical resistance, high corrosion resistance, lightweight, non-conductivity, etc. FRP rebar is anisotropic material that can be manufactured by either a pultrusion process or braiding technique. The pultrusion process is inexpensive and can rapidly produce a member with a constant cross-section. Composites produced by the pultrusion process are good in structural applications due to their continuous mass production with homogeneous mechanical properties. However, because members generated by the pultrusion process have a smooth surface, an additional step of digging or excavating with a machine and protruding or coating the surface is needed to increase the strength of its bond with the concrete interface. The braiding technique is a modification of the pultrusion process that creates protrusions via a weaving process prior to the hardening stage. Although this braiding method is difficult in practice and the fiber content is less than in the pultrusion process, external protrusions can be created easily. The braiding technique was used to manufacture the carbon fiber-reinforced polymer (CFRP) rebar specimens in this study. In order to improve the adhesion performance of the FRP rebar, surface treatment of the protrusion was preferred. Among FRPs, the CFRP has higher tensile strength and elastic modulus than steel rebar. Based on the previous results, the mechanical performance of the FRP rebar used in construction and civil engineering was verified, and the CFRP rebar was confirmed to be used as a steel rebar substitute [1,2]. Additional studies are needed to better understand the structural behavior of FRP concrete structures. First, the mechanical properties, which include the tensile strength, elastic modulus, and shear strength, of FRP rebar must be determined. Test standards for FRP rebar already have been established in the United States and Canada. In 2006, the American Concrete Institute (ACI) 440 Committee presented the specification 440.1R-06 and published a revised version of ACI 440.1R-15 in 2015 [2,3]. The ACI 440.3R-12 [4] specifies a test method for FRP rebar applied to structures and concrete. The test methods include those for mechanical properties, such as tensile strength, adhesion, creep, and flexure properties. In Canada, a specification referred to as the Design and Construction of Building Structures with Fibre-Reinforced Polymers (CHBDC CAN/CSA-S6-06) was proposed in 2006, and CHBDC CAN/CSA-S6-14 and CAN/CSA S806-12 have been proposed more recently [5,6,7]. In Korea, KS F ISO 10406-1 was enacted in 2017 as a test standard for FRP rebar [8]. However, the existing standards have not been fully developed for large diameters of CFRP rebars. Several studies have been conducted over the past decades and available findings are reviewed herein.

Benmokrane et al. [9] successfully completed the tensile test for CFRP rebar (less than ϕ8 mm). He reported that the pull-out behavior is affected by the surface geometry of FRP rods, the properties of the grout, and the stiffness of the anchoring tube. Khan et al. determined the mechanical properties of glass fiber-reinforced polymer (GFRP) rebar (ϕ15.9 mm) and CFRP rebar (ϕ15 mm) in accordance with ASTM D7205 (tension test) and ASTM D695 (compression test) [10]. Khan et al. reported that the modulus of elasticity of CFRP rebar is greater than that of GFRP rebar, although the tensile strength of CFRP rebar is less than that of GFRP rebar due to the lower percentage of CFRP fibers by volume than the GFRP rebars [11]. Plevkov et al. determined the tensile and compressive strength values of CFRP rebar (ϕ10 mm and GFRP rebar (ϕ10 mm) and reported that CFRP rebar has greater tensile strength and a higher elasticity modulus value than GFRP rebar. Plevkov et al. also reported crushing failure at the end tips, so they devised a compressive strength test that involves fitting steel caps to the ends of GFRP rebar and then filling them with concrete [12]. Koosha and Pedram [13] introduced a new test method to determine the compressive properties of GFRP rebar. GFRP rebar with diameters of 13 mm, 16 mm, and 19 mm were used. Steel caps were attached to both ends of the specimen to prevent alignment of the specimen and premature failure at the end of the specimen. In addition, two gauges were installed on the specimen to conduct a compression test. AlAjarmeh et al. [14] proposed a new test for compressive properties by mounting a hollow steel cap on both ends of a GFRP rebar and filling it with cement grout because there was no test method for compressive strength test of GFRP. GFRP rebars with diameters of 9.5 mm, 15.9 mm, and 19.1 mm were used, and the length of the specimen was set to diameter ratios of 2, 4, 8, and 16. Only a few test results are available for the CFRP rebars.

In short, as addressed above, the fundamental mechanical characteristics of the CFRP bars, including the tensile strength, compressive strength, shear strength, and modulus of elasticity of the CFRP bar, need to be accurately determined to understand the behavior of the concrete structures reinforced with CFRP rebars. In addition, the bar size effects on the mechanical properties of CFRP rebars are investigated in this study.

## 2. Testing of Mechanical Properties of CFRP Rebar Specimens

### 2.1. Materials

**Figure 1:**
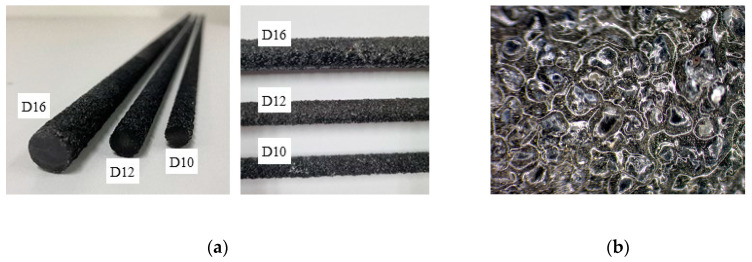
Sand-coated carbon fiber reinforced polymer rebar specimens with three different diameters. (**a**) Various sizes of CFRP rebar; (**b**) Surface of sand-coated CFRP rebar.

The FRP rebar has lower adhesion to concrete than steel rebar [15]. The CFRP rebar specimens used in this study were sand-coated to improve the bond strength of the concrete. Figure 1 shows the sand-coated CFRP rebar specimens with diameters of 10 mm, 12 mm, and 16 mm. The manufacturer (SK chemical, Seoungnamsi, Korea) provided the following information regarding the properties: ultimate stress > 2850 MPa (based on ASTM D3039M [16]), modulus of elasticity > 158 GPa, and ultimate strain > 1.8 percent. The ratio of carbon fiber (CF) to total area is about 42%.

### 2.2. Tensile Strength Testing of CFRP Rebar Specimens

The tensile strength tests of the CFRP rebar specimens were conducted in accordance with ASTM D7205 [17]. Five CFRP rebars for each of the three diameters, D10, D12, and D16 were prepared for the tensile strength test. The ASTM D7205 standard specifies the tensile strength test method suggested in ACI 440.3R-12 [4]. This test can determine the tensile strength of FRP matrix composite rebar that typically is used as a tensile element in rebar and prestressed post-tension concrete. Table 1 presents the dimensions of the tensile strength test CFRP rebar specimens used in this study. A steel tube (thickness: 2 mm) filled with epoxy at both ends of the specimen was fabricated in accordance with ASTM D7205. Due to the length limitation of testing equipment, the specimen for D16 was designed with a grip length of 660 mm.

**Figure 2:**
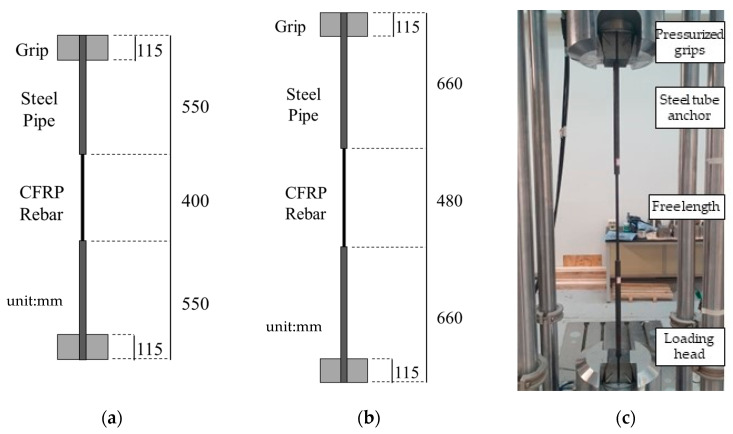
Tensile strength test of CFRP rebar based on ASTM. (**a**) dimensions for CFRP D10 rebar specimen; (**b**) dimensions for CFRP D12 and D16 rebar specimens; (**c**) tensile strength test set-up D7205 (at Intelligent Construction System Core-Support Center, Keimyung University).

Figure 2a schematically presents the CFRP D10 rebar specimen fabricated for the tensile strength test. Figure 2a presents the CFRP D10 rebar specimens fabricated for the tensile strength test with a grip length of 550 mm, free length of 400 mm, and total length of 1500 mm. Figure 2b schematically presents the CFRP D12 and D16 rebar specimens fabricated for the tensile strength test with a grip length of 660 mm, free length of 480 mm, and total length of 1800 mm. Figure 2c presents the tensile strength test set-up whereby the load is applied in displacement control mode using a universal testing machine (UTM) with a capacity of 1200 kN at the rate of 3 mm/min.

### 2.3. Compressive Strength Testing of CFRP Rebar Specimens

**Figure 3:**
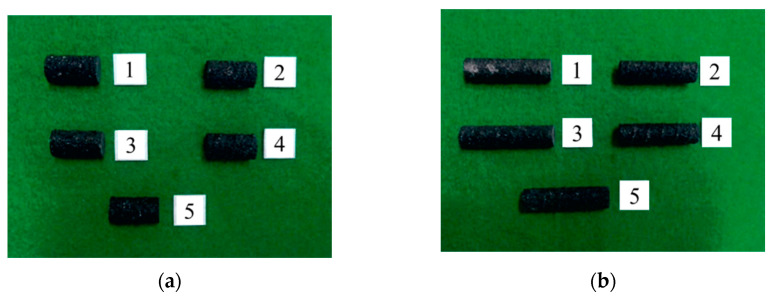
Compressive strength test specimens (D12). (**a**) Specimen length two times its diameter; (**b**) specimen length four times its diameter (for modulus of elasticity tests).

The compressive strength tests were conducted using CFRP rebar specimens with their lengths set to two times the diameter of the specimen in accordance with the compressive strength test method specified in ASTM D695 [18]. Five specimens for each of the three diameters, D10, D12, and D16, were prepared for each compressive strength test. Modulus of elasticity tests were conducted using specimens with their lengths set to four times the diameter of the specimen, as shown in Figure 3. Table 2 provides the dimensions of the compressive strength test CFRP rebar specimens. Note that ‘2D’ and ‘4D’ refer to two times and four times the diameter, respectively.

**Figure 4:**
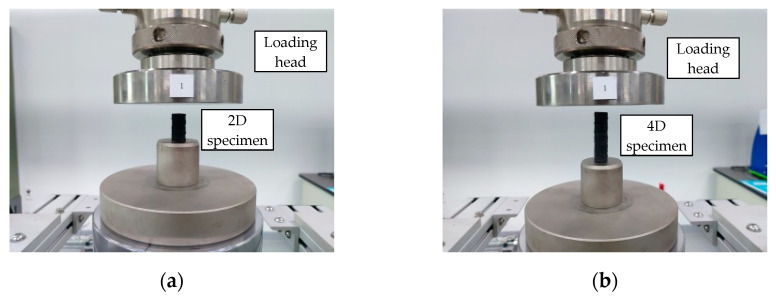
Compressive strength test set-up based on ASTM D695. (**a**) D12 (length 2 times diameter) test set-up; (**b**) D12 (length 4 times diameter).

Figure 4a,b show identical compressive strength test set-ups for the specimens with their lengths two times and four times their diameters, respectively. The load was applied in displacement control mode at a rate of 1 mm/min using a 100-kN UTM.

### 2.4. Shear Strength Testing of CFRP Rebar Specimens

The shear strength tests were conducted using five specimens for each of the three diameters, D10, D12, and D16. 

**Figure 5:**
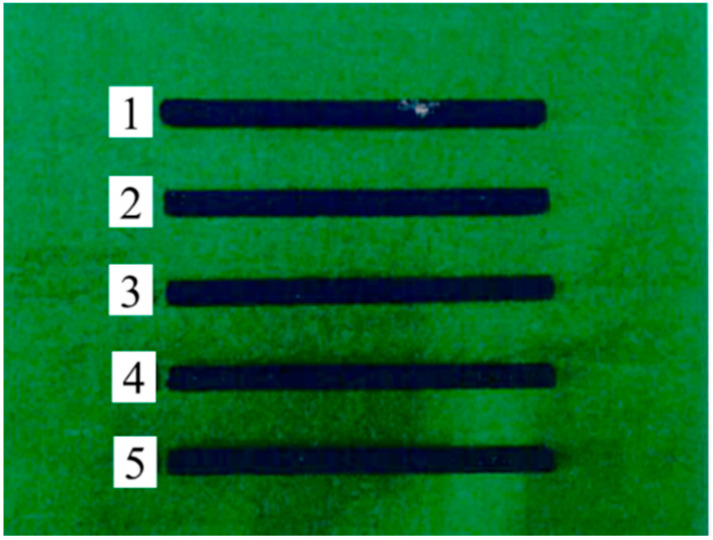
Five specimens used for shear strength tests.

Figure 5 shows the test specimens that were fabricated with the length of 225 mm in accordance with ASTM D7617 [19]. The set-up required for this test was designed specifically to fit the specimen for each diameter according to the ASTM D7617 standard.

**Figure 6:**
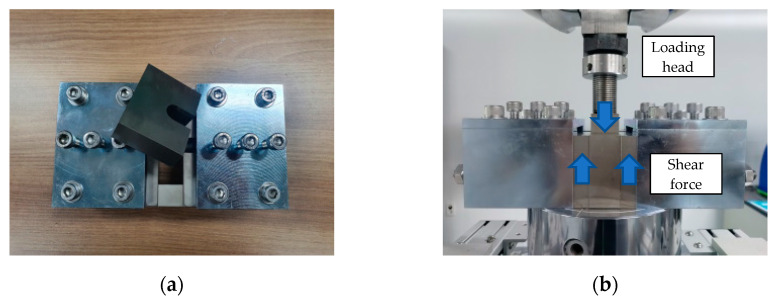
Shear strength test of CFRP rebar based on ASTM D7617. (**a**) shear jig; (**b**) test set-up.

Figure 6a shows the shear jig used for the shear strength test and Figure 6b shows the shear strength test set-up. The load was applied in displacement control mode at the rate of 1 mm/mm using a 100-kN UTM.

## 3. Test Results and Discussion

### 3.1. Tensile Strength Test Results

**Figure 7:**
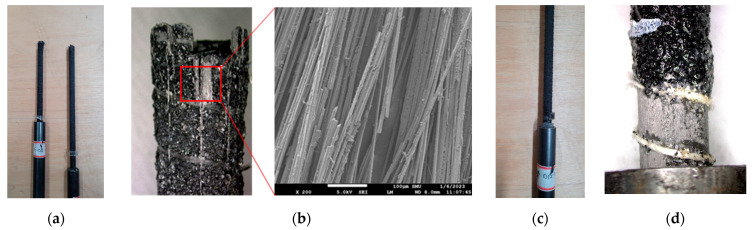
Tensile strength test specimens and failure location or mode. (**a**) center; (**b)** central failure mode; (**c**) grip; (**d**) grip failure mode.

Figure 7 shows the tensile strength test specimens and the location and mode of failure. Figure 7a,c show that the CFRP rebar specimens fractured at the center and grip of the specimens, respectively. Figure 7b shows the fracture of the CFRP fiber at the center and that fiber weave is unidirectional. As the fiber weave is generated in one direction, the fibers of the CFRP rebar break sequentially, thus resulting in a brittle fracture. Figure 7d shows a fracture at the grip of the specimen.

Table 3 presents the tensile strength test results for the CFRP rebar specimens in terms of tensile strength value, modulus of elasticity value, and failure mode. Table 3 also provides the average values of the tensile strength and modulus of elasticity.

**Figure 8:**
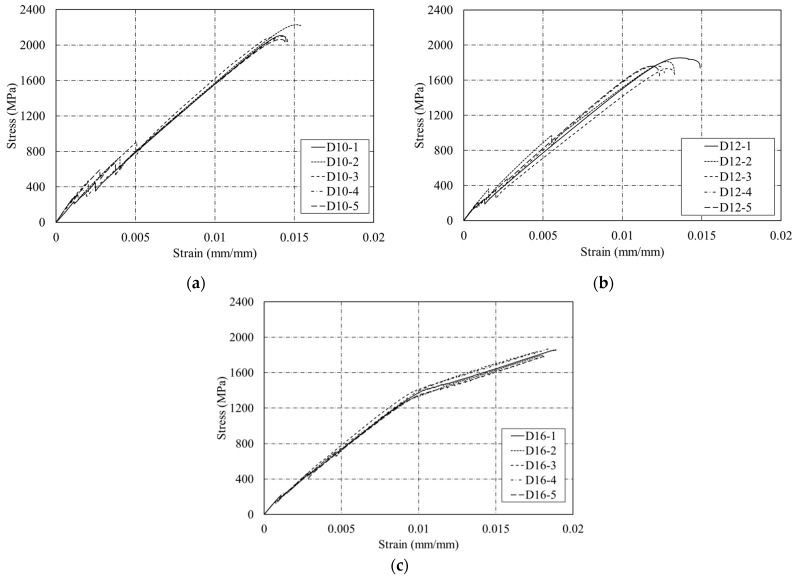
Stress–displacement relationship of five sand-coated CFRP rebar specimens. (**a**) D10; (**b**) D12; (**c**) D16.

Figure 8a–c present the stress–displacement relationship of the D10, D12, and D16 CFRP rebar specimens, respectively. The results clearly indicate that CFRP rebar has no yield point. For the D16 specimens, an unexpected bilinear relationship was observed due to the slippage at the grip area. The average tensile strength value of the five D12 specimens tested is 1784 MPa. This value satisfies the standard tensile strength range of 600 MPa to 3690 MPa for CFRP rebar specified in ACI 440.1R-15 [2]. The average modulus of elasticity value is 158 GPa, which is also within the standard modulus of elasticity range of 120 GPa to 580 GPa for CFRP rebar specified in ACI 440.1R-15. Moreover, these results also satisfy the standard (KS F ISO 10406-1) modulus of elasticity value for CFRP rebar because the design guidelines (KS F ISO 10406-1) specify 140 GPa for FRP rebar. In addition, tensile strength tests of the five D10 and five D16 specimens show tensile strength values of 2116.7 MPa ± 58.47 MPa for D10 and 1831.3 MPa ± 33.19 MPa for D16.

### 3.2. Compressive Strength Test Results

**Figure 9:**
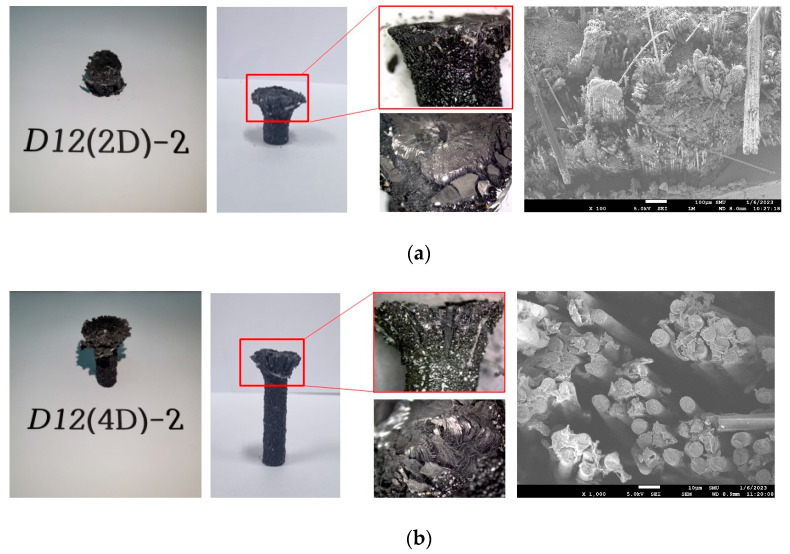
Compressive strength test specimens and their failure modes. (**a**) D12 (length 2 times diameter); (**b**) D12 (length 4 times diameter).

Figure 9a,b present photos of compressive strength test D12 specimens with lengths that are two times and four times their diameters, respectively, and their failure modes. All the CFRP D12 rebar specimens were crushed at the point where the load was applied, as shown in the figures. The fibers became separated from each other due to the failure of the resin rather than buckling.

Table 4 presents the results of the compressive strength tests for each of the five specimens with each of the three diameters (D10, D12, and D16) and for each of the lengths (two times and four times the diameter, respectively). The compressive strength test results of the CFRP rebar indicate an average compressive strength of 357 MPa in the case of the D12 CFRP rebar. The compressive strength of the CFRP rebar specimen with a length that is twice the diameter is approximately 7% greater than that of the CFRP rebar specimen with a length that is four times the diameter. In addition, the compressive strength values of the CFRP rebar with lengths that are twice and four times the diameter are 79.9% and 81.2% lower than the tensile strength, respectively. The results of the compressive strength tests of the CFRP rebar indicate that the average compressive strength values of D10 and D16 CFRP rebar are 399 MPa and 360 MPa, respectively. The compressive strength of the CFRP D10 rebar specimen with a length that is twice the diameter is approximately 12% greater than that of the CFRP D10 rebar specimen with the length that is four times the diameter, whereas the compressive strength of the CFRP D16 rebar specimen with the length that is twice the diameter is approximately 9% smaller than that of the CFRP D 16 rebar specimen with the length that is four times the diameter.

### 3.3. Shear Strength Test Results

**Figure 10:**
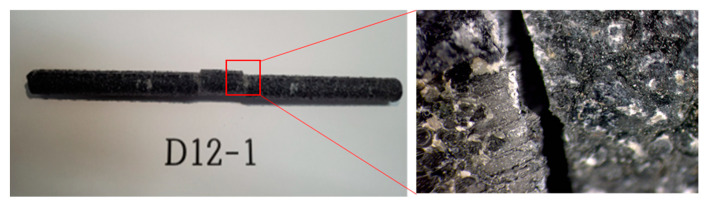
Typical failure mode for shear strength test of D12 Specimen.

Shear strength tests of five specimens for each of the three diameters (D10, D12, and D16) were conducted, and Figure 10 shows a photo of the typical shear failure observed for the D12 specimens.

Table 5 presents a summary of the shear strength test results for the five specimens for each of the three diameters. The results of the shear strength tests of the CFRP rebar indicate that the average shear strength values of D10, D12, and D16 CFRP rebar are 371 MPa, 360 MPa, and 283 MPa, respectively.

**Figure 11:**
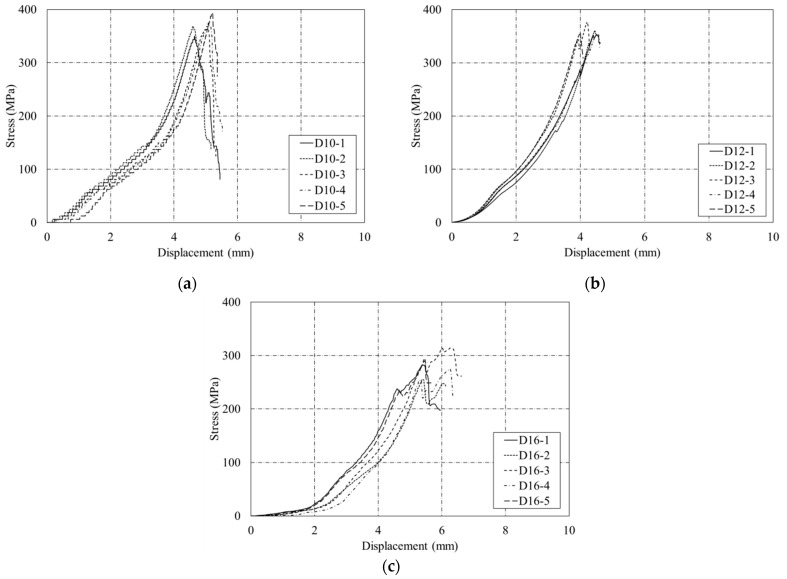
Stress–displacement relationship of five shear strength test specimens. (**a**) D10; (**b**) D12; (**c**) D16.

Figure 11 shows the stress–displacement relationships of five shear strength test specimens for each of the three dimensions. Figure 11a shows that the stress increased up to 371 MPa for the CFRP rebar D10. Figure 11b shows that the stress of the CFRP rebar D12 test specimens continuously increased until failure. The CFRP rebar D16 had smaller shear strength than CFRP rebars D10 and D12, as shown in Figure 11c. According to the results of shear strength tests of FRP in previous studies [20], CFRP rebar maintains constant stress before failure and exhibits failure in terms of horizontal and vertical cracks. However, by contrast, the shear strength test results obtained in this study indicate that CFRP rebar shows a tendency to fracture immediately without resistance to a constant load. The reason for this outcome appears to be due to the FRP weaving method. The CFRP specimens used in this study showed significant resistance to loading in the longitudinal direction because they were fabricated in one direction and thus were vulnerable to shear. Therefore, specimens should be fabricated based on three dimensions instead of using single-directional weaving methods to improve the shear performance of CFRP rebar.

## 4. Discussion

### 4.1. Effect of Size of CFRP Rebar

**Figure 12:**
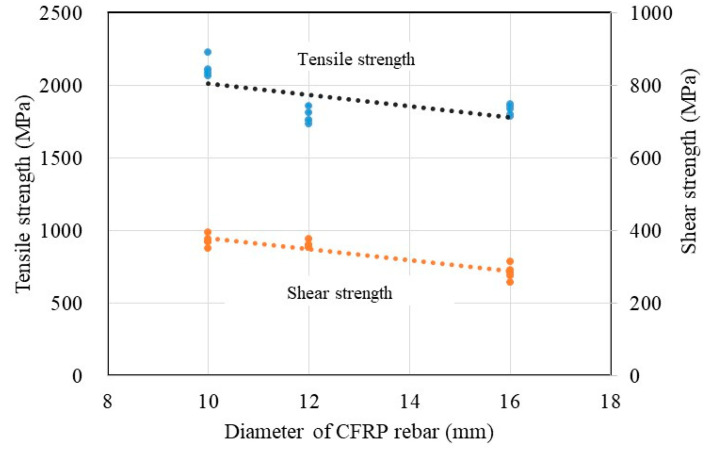
Effect of bar size for tensile strength and shear strength.

With an increase in the diameter of CFRP rebar, the tensile strength tends to decrease due to an uneven tensile stress distribution throughout the cross-section. This result is matched well with the experimental results in the literature [21]. Furthermore, unlike steel rebar, CFRP rebar has an orientation due to its fiber inclusion, and both the strength and stiffness of CFRP rebar vary according to the fiber content and resin used. Therefore, the tensile strength tests should be conducted using many specimens in order to ensure the reliability of the material’s mechanical performance. Similarly, the shear strength is significantly affected by the size of CFRP rebar due to the fiber matrix and resin. The shear strength is reduced as an increase in the CFRP rebar size as shown in Figure 12. Regardless of the CFRP rebar size, the tensile modulus of elasticity of CFRP rebars was higher than their compressive modulus of elasticity. The results are matched well with the findings in the literature [10]. 

Jung et al. [22] reported the modulus of elasticity of hybrid rebar to be approximately 100 GPa. The CFRP D12 rebar investigated in this study showed a modulus of elasticity value that is 1.58 times higher than that of the hybrid rebar but lower than that of steel rebar (200 GPa). Plevkov et al. [12] found that the modulus of elasticity value of general CFRP rebar is 144 GPa, which is 0.91 times lower than that of the CFRP D12 rebar used in this study. Therefore, the CFRP rebar produced in the future as steel rebar replacement should have enhanced strength, which can be accomplished by conducting additional research studies using different fiber arrangements/orientations and different resin contents.

### 4.2. Relationship between Shear Strength and Tensile Strength

As addressed in the literature [10], the tensile test for the CFRP rebar needs high attention to avoid premature failure in tension. As increasing CFRP rebar size, the test setup required a large free length, enough steel pipe anchor, and a high-capacity loading machine. Simply, a correlation between tensile strength and shear strength can be employed to predict the tensile strength of CFRP rebar according to the rebar’s diameter. Equation (1) can be used to calculate the ratio of shear strength to tensile strength using the results of shear and tensile strength tests of CFRP rebar with a diameter up to 16 mm [21].

(1) $$k_{d}=\frac{\sum \frac{f_{s}}{f_{t}}}{n_{t}},$$

where *k_d_* is the coefficient; *f_s_* is the shear strength; *f_t_* is the tensile strength.

**Figure 13:**
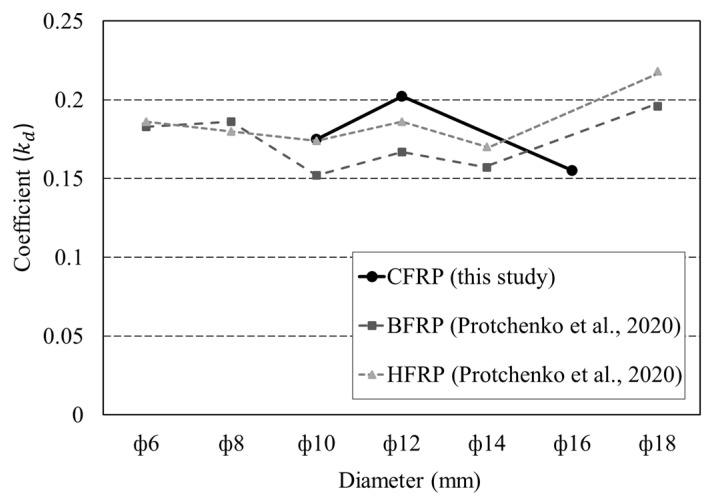
Calculated coefficients with respect to rebar diameter for sand-coated CFRP, basalt FRP, and hybrid FRP [21].

Figure 13 shows the computed coefficients (*k_d_*). The literature shows that these coefficients (*k_d_*) range from 0.163 to 0.207 for basalt FRP rebar and basalt/CFRP hybrid rebar depending on the diameter of the rebar [21]. The values obtained in this study match well with the results from the literature. Therefore, the calculated coefficients (*k_d_*) can be used to predict the tensile strength of CFRP D10 to D16 rebar.

## 5. Conclusions

Tensile, compressive, and shear-strength tests were conducted in this study in accordance with the test methods specified in ASTM and other international standards to evaluate the mechanical performance of CFRP rebar. As a result of the tests and quantification of the mechanical performance of the CFRP rebar specimens, the following conclusions can be drawn.

The results of the tensile strength tests of CFRP rebar conducted in accordance with ASTM D7205 indicate that the average tensile strength value is 1784 MPa and the average modulus of elasticity value is 158 GPa. This value satisfies the standard modulus of elasticity values specified in ACI 440.1R-15 (in the range of 120 GPa to 580 GPa) and the minimum modulus of elasticity value of 140 GPa specified in guidelines for the structural design of FRP rebar. 

For the compressive strength and modulus of elasticity tests, the test results indicate that the average compressive strength value is 357 MPa and the average modulus of elasticity value is 30 GPa. The tensile modulus of elasticity of CFRP rebars was higher than their compressive modulus of elasticity.

As the results of the shear strength test, all specimens are continuously increased until failure. In short, the shear strength and tensile strength of CFRP rebar are affected by the CFRP rebar size. In addition, as a result of investigating the relationship between the shear strength and tensile strength of CFRP, it was possible to predict the coefficient according to the diameter of the CFRP rebar.

Based on the mechanical performance testing, the sand-coated CFRP rebar was determined in this study. The following study will use this information for further structural tests and analyses. To achieve the target tensile strength value of 2100 MPa in future research, the resistance to tension should be increased throughout the polymer section by changing the fiber arrangement/orientation of the CFRP rebar to improve its brittle-resistant properties. In addition, to prevent grip failure and slippage, an adequate bond system along with ASTM specification is needed for future tests.

## Figures and Tables

**Table 1:** Dimensions of CFRP rebar specimens used for tensile strength tests.

Diameter (mm)	Grip Length (mm)	Free Length (mm)	Total Length (mm)
10	550	400	1500
12	660	480	1800
16	660	480	1800

**Table 2:** Dimensions of CFRP rebar specimens used for compressive strength tests.

Specimen	Diameter (mm)	Height (mm)
D10 (2D)	10	20
D10 (4D)	10	40
D12 (2D)	12	24
D12 (4D)	12	48
D16 (2D)	16	32
D16 (4D)	16	64

**Table 3:** Tensile test results.

Specimen	Tensile Strength (MPa)	Tensile Modulus of Elasticity (GPa)	Failure Mode
D10-1	2107	161	Grip failure
D10-2	2229	144	Grip failure
D10-3	2083	153	Grip failure
D10-4	2100	158	Grip failure
D10-5	2062	133	Grip failure
Average	2116 ± 58.50	150 ± 10.19	
D12-1	1854	152	Grip failure
D12-2	1811	173	Grip failure
D12-3	1732	144	Center failure
D12-4	1762	162	Grip failure
D12-5	1758	158	Grip failure
Average	1784 ± 43.57	158 ± 9.72	
D16-1	1859	136	Grip failure
D16-2	1799	135	Specimen end
D16-3	1839	145	Grip failure
D16-4	1871	136	Grip failure
D16-5	1786	131	Grip failure
Average	1831 ± 33.16	136 ± 4.59	

**Table 4:** Compressive test results.

Description			1	2	3	4	5	Average
D10	(2D)	*f_comp._* (MPa)	402	452	427	314	402	399
	(2D)	Compressive modulus of elasticity *E_comp_* (GPa)	14	17	15	13	15	15
	(4D)	*f_comp._* (MPa)	369	355	314	333	408	356
	(4D)	Compressive modulus of elasticity *E_comp_* (GPa)	29	26	23	23	30	26
D12	(2D)	*f_comp._* (MPa)	350	392	420	344	277	357
	(2D)	Compressive modulus of elasticity *E_comp_* (GPa)	16	16	16	13	13	15
	(4D)	*f_comp._* (MPa)	356	311	335	398	273	335
	(4D)	Compressive modulus of elasticity *E_comp_* (GPa)	31	30	28	36	26	30
D16	(2D)	*f_comp._* (MPa)	326	348	332	410	382	360
	(2D)	Compressive modulus of elasticity *E_comp_* (GPa)	13	18	17	19	18	17
	(4D)	*f_comp._* (MPa)	453	429	321	403	368	395
	(4D)	Compressive modulus of elasticity *E_comp_* (GPa)	42	38	34	46	41	40

**Table 5:** Shear strength test results.

Description	D10	D12	D16
Shear Strength (MPa)	350	353	282
	368	360	256
	368	376	314
	375	353	275
	393	357	290
Average	371	360	283

## Data Availability

Not applicable.

## References

[1] Cho J.R., Park Y.H., Park S.Y., Park C.W. (2018). Development of Design Guidelines for FRP Reinforced Concrete Structure and Application of FRP Reinforcement in Korea. Mag. Korea Concr. Inst..

[2] (2015). Guide for the Design and Construction of Structural Concrete Reinforced with Fiber-Reinforced Polymer (FRP) bars.

[3] (2006). Guide for the the Design and Construction of Structural Concrete Reinforced with fiber-Reinforced Polymer (FRP) bars.

[4] (2012). Guide for the Design and Construction of Structural Concrete Reinforced with Fiber-Reinforced Polymer (FRP) Bars.

[5] (2006). Canadian Highway Bridge Design Code.

[6] (2014). Canadian Highway Bridge Design Code.

[7] (2012). Design and Construction of Building Structures with Fibre-reinforced Polymers.

[8] (2017). Fibre-Reinforced Polymer (FRP) Reinforcement of Concrete-Test Methods-Part 1: FRP Bars and Grids.

[9] Benmokrane B., Zhang B., Chennouf A. (2000). Tensile Properties and Pull out Behaviour of AFRP and CFRP rods for grouted anchor applications. Constr. Build. Mater..

[10] Khan Q.S., Sheikh M.N., Hadi M.N.S. Tension and Compression Testing of Fibre Reinforced Polymer (FRP) Bars. Proceedings of the 12th International Symposium on Fiber Reinforced Polymers for Reinforced Concrete Structures (FRPRCS-12) & the 5th Asia-Pacific Conference on Fiber Reinforced Polymers in Structures (APFIS-2015) Joint Conference.

[11] Khan Q.S., Sheikh M.N., Hadi M.N.S. (2021). Tensile Testing of Carbon FRP (CFRP) and Glass FRP (GFRP) Bars: An Experimental Study. J. Test. Eval..

[12] Plevkov V., Baldin I., Kudyakov K., Nevskii A. (2017). Mechanical Properties of Composite Rebar under Static and Short-term Dynamic Loading. AIP Conf. Proc..

[13] Koosha K., Pedram S. New Testing Method of GFRP Bars in Compression. Proceedings of the CSCE Annual Conference 2018.

[14] Ashrafi H., Bazli M., Najafabadi E.P., Oskouei A.V. (2017). The Effect of Mechanical and Thermal Properties of FRP bars on their Tensile Performance under Elevated Temperatures. Constr. Build. Mater..

[15] Lee S.T., Park K.P., Park K.T., You Y.J., Seo D.W. (2019). A study on the Application of FRP Hybrid Bar to Prevent Corrosion of Reinforcing Bar in Concrete Structure. J. Korea Acad. Ind. Coop. Soc..

[16] (2014). Standard Test Method for Tensile Properties of Polymer Matrix Composite Materials.

[17] (2016). Standard Test Method for Tensile Properties of Fiber Reinforced Polymer Matrix Composite Bars.

[18] (2010). Standard Test Method for Compressive Properties of Rigid Plastics.

[19] (2017). Standard Test Method for Transverse Shear Strength of Fiber-reinforced Polymer Matrix Compo-site Bars.

[20] Park C.G., Won J.P. (2003). Mechanical Properties of Hybrid FRP Rebar. J. Korean Soc. Agric. Eng..

[21] Protchenko K., Zayoud F., Urba´nski M., Szmigiera E. (2020). Tensile and Shear Testing of Basalt Fiber Reinforced Polymer (BFRP) and Hybrid Basalt/Carbon Fiber Reinforced Polymer (HFRP) Bars. Materials.

[22] Jung K.S., Park K.T., Seo D.W., Kim B.C., Park J.S. (2017). Prediction of Tensile Behavior for FRP Hybrid Bar. J. Korean Soc. Adv. Compos. Struct..

